# Laryngeal metastasis of a prostate carcinoma: one rare entity

**DOI:** 10.1590/S1808-86942012000300023

**Published:** 2015-10-14

**Authors:** José Alberto Alves Oliveira, Roberta de Almeida Said, Rafaella de Sousa Cartaxo, José Alexandre Macedo dos Santos, Ricardo Lincoln Pinto Gondim

**Affiliations:** a Medical student of the 6th year - State University of Ceará.; b MD. Radiologist - Cancer Hospital/Cancer Institute of Ceará.; c MD. Radiologist resident, Cancer Hospital/Cancer Institute of Ceará.; d MD. Radiologist - Cancer Hospital/Cancer Institute of Ceará.; e MD. Head and Neck Surgeon - Cancer Hospital/Cancer Institute of Ceará and Fortaleza General Hospital.

**Keywords:** larynx, neoplasm metastasis, prostatic neoplasms

## INTRODUCTION

Prostate cancer is the most common neoplasia affecting men. The larynx is an uncommon site for metastasis; there is a very limited number of reports on this topic. Melanomas and renal carcinomas are the most common types of primary tumors which metastasize to the larynx, followed by breast, lungs and colon cancer^1^, ^2^, ^3^, ^4^. Laryngeal metastatic involvement by prostate cancer is a rare finding in clinical practice^1^, ^2^.

## CASE PRESENTATION

A 73-year-old patient was diagnosed in May, 2008, with Gleason 7(3+4) prostate adenocarcinoma, with an initial PSA of 78 ng/mL and bone involvement found upon bone scintigraphy. Treatment started with goserrelin and bisphosphonate.

**Figure 1:**
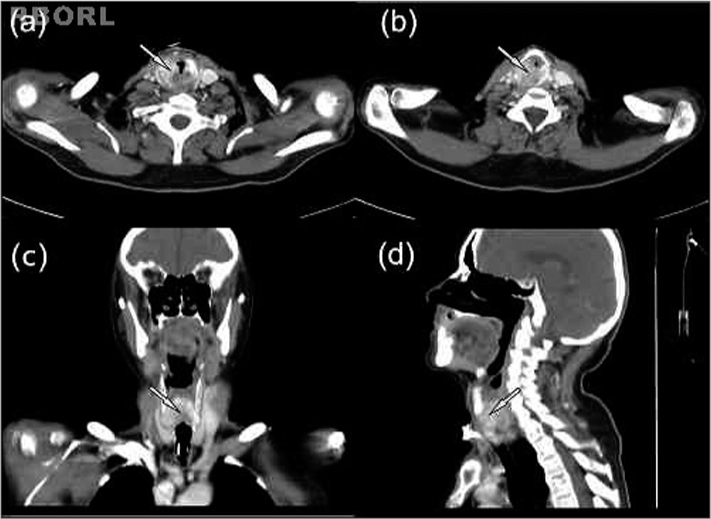
Heterogeneous volumetric enlargement of the cricoid cartilage, with demineralization and erosion associated to soft tissue involvement, causing an important stenosis of the laryngeal airway (arrows). (a and b): craniocaudal sequential axial view; (c): posterior coronal view; (d): paramedian sagittal view.

After one month, he started complaining of hoarseness. The video-laryngoscopy showed right-side vocal cord paralysis associated with laryngitis. His PSA level dropped to 9.1 ng/mL after three months of treatment; notwithstanding, he kept complaining of hoarseness. He was submitted to a neck CT scan, which showed the lesion involving the entire cricoid cartilage (Figure 1). A laryngeal biopsy was suggested, but the patient refused it.

After an additional PSA drop to 3.8 ng/mL, in November of 2008, the patient chose to interrupt the hormone-therapy, against medical advice. Five months later, he started to complain of intense pain on the left shoulder, worsening in his hoarseness and noisy breathing. He developed respiratory obstruction, which led to an urgent tracheostomy. He was also found with a pathological fracture in his left humerus, which required surgery. In the same procedure, he was finally submitted to the cricoid biopsy. The specimen histology proved to be carcinoma, and immunohistochemical assays showed the expression of low-weight cytokeratin and PSA, but no p63 or CK5/6. The result diagnosis was metastasis of a prostatic cancer. The patient was referred to radiotherapy on his left humerus and neck, and he is currently under treatment with an LHRL analogue, peripheral antiandrogen and zoledronic acid.

## DISCUSSION

Laryngeal metastatic cancers are rare. Notwithstanding, this organ must not be discarded as a potential metastatic target. The tumors which most often metastasize to the larynx are the melanoma and the clear cell renal carcinoma. There are, however, reports of colon, pancreas, breast and prostate cancer metastases^1^, ^3^. The supraglottis is the most affected site by these metastases, and the glottis is the less frequent one^3^.

Laryngeal involvement by the tumor may or may not cause symptoms, hoarseness and stridor. A careful initial ENT physical exam may detect lesions which can still be operated upon and prevent the sudden and catastrophic airway obstruction. Laryngeal CT scan is important to outline the lesion to the larynx and to define neck lymph node involvement. A visible tumor mass seen upon laryngoscopy implies the need to biopsy the lesion and send it to a pathology exam^4^.

There are only 13 reports in the literature concerning the laryngeal metastasis of prostate origin. *Post mortem* exams showed that the incidence of such event seems to be higher that clinical experience suggests; among six patients with metastatic prostate cancer submitted to an autopsy with laryngeal anatomical sections, all had unsuspected laryngeal involvement. However, the common lack of local growth and the scarcity of laryngeal symptoms, seem to be limiting factors for diagnosis, as well as laryngeal involvement being found in advanced stages of the disease in terminal patients^1^.

## FINAL REMARKS

Patients with a past of prostate, kidney, breast, colon cancer or malignant melanoma, with symptoms of hoarseness and sore throat, must be investigated as to the possible presence of a metastatic laryngeal cancer.

The present study aimed at contributing with the current literature on the topic at hand, having seen the few number of cases reported on the topic.
